# Attempted suicide and pregnancy in COVID-19´s times

**DOI:** 10.1192/j.eurpsy.2021.725

**Published:** 2021-08-13

**Authors:** M.O. Solis, L. Soldado Rodriguez, S.S. Sánchez Rus, M. ValverDe Barea, S. Jimenez Fernandez

**Affiliations:** 1 Jaén, Complejo Hospitalario Jaén, Jaén, Spain; 2 Mental Health Unit, Complejo Hospitalario de Jaen, Jaen, Spain

**Keywords:** COVID-19, Depression, Suicide, pregnancy

## Abstract

**Introduction:**

Pregnancy is a special risk factor for suicide attempts among females (Andrew E. Czeizel et al. 2011). Situational factors such as the novel coronavirus (COVID-19) have also been reported to impact on individual’s mental health.

**Objectives:**

Examine the effect of COVID-19 and its association with mental health and attempt suicide risk in pregnant population.

**Methods:**

A cross-sectional study that includes 113 pregnant women from Spain, through an anonymous, voluntary and multiple response type online survey which included questions about socio demographic aspects, COVID-19’s aspects and mental health.

**Results:**

Of the 112 pregnant patients surveyed, only 2 reported suicidal ideation. The age of the respondents was 32 and 33 years, both of whom were in the 2nd trimester of pregnancy. Both report that it was the first pregnancy and affirm a worsening of their economic situation since the beginning of the COVID-19 pandemic. One of them did not have a partner / marriage and was living with a relative, in turn this respondent was unemployed. Both responded that they were “always” worried about the possible outcome of the COVID-19 pandemic and that their fears had increased, being difficult to control and let them pass. It is very relevant that both agree that they “never” felt that the professionals who carried out the pregnancy controls asked or inquired about their current state of mental health.

**Conclusions:**

Antepartum suicidal ideation is an important and common complication of pregnancy, specially in COVID-19’s times, healthcare professionals who follow pregnancy should detect high-risk suicidal patients and be able to carry out a suicide prevention program.

